# Letter to the editor regarding “developing a framework for medical student feedback literacy using a triangulated thematic analysis”. A UK student perspective

**DOI:** 10.1080/07853890.2026.2728178

**Published:** 2026-09-26

**Authors:** Ahmed Abbas, Shraya Pandya, Noreen Ryan

**Affiliations:** School of Public Health, Imperial College London, London, UK

Dear Editor

I had the pleasure of reviewing the paper ‘Developing a framework for medical student feedback literacy using a triangulated thematic analysis’ by Noor et al. [1] As an Imperial College London medical student, I would like to share my perspectives within the context of the UK education system. 

The study implemented a feedback framework integrating patient safety and reflective practice. This framework was compared to two established feedback models [2,3] focusing on seven core themes including: foundations, diverse sources, engagement, analysis, evaluation, response and internalisation of feedback. They have proposed expanding this framework internationally, where I feel this will be practical in the UK, given certain adaptations-particularly to reflect the multidisciplinary culture of the NHS, the timetabling pressures faced by medical schools, and the limited availability of lecturers to provide timely feedback.

 The Malaysian culture has been described as ‘hierarchical’ [1]. In the UK, this is slowly diminishing with greater emphasis on multi-disciplinary teams in medical management  [4,5].UK medical students encounter and receive feedback from a wide variety of health professionals. Moreover, formative assessments within UK medical schools involve receiving feedback based on examinations performed and history taken, and the NHS culture of mandatory quality improvement projects further reinforces expectations of feedback. This combination may make the theme 2 result - preparing for real-time feedback from diverse sources- [1] particularly relevant in the UK.

Theme 3 highlights the importance of active interactions between students and lecturers[1].This would be ideal in the UK, given NHS practice requires incorporating feedback from senior members of staff. However, practical barriers exist. Consistent contact with the same lecturers is uncommon, as students rotate frequently, and many educators are part-time teachers who balance clinical or research roles. This limits opportunities to build rapport and reduces the feasibility of providing tailored feedback. Combined with a crowded curriculum [6], scheduling pressures, and declining numbers of clinical academics in UK medical schools, implementing a system reliant on sustained lecturer–student interaction may be challenging. Any adaptation of the framework must therefore consider resource constraints.

 Theme 3 also highlights students should know how to provide good feedback [1]. This aligns closely with UK educational priorities, given the GMC Outcomes for Graduates framework stipulates the importance of giving and receiving feedback [7]. Within Imperial, various feedback models are not only taught but assessed in summative exams. This may incentivise students to engage in feedback processes -such as the Malaysian study approach- as by learning the theory of feedback, students are more likely to appreciate why it is effective beyond the realms of simply being told by a lecturer or senior.  

In summary, this review highlights the potential value of applying a Malaysian feedback framework to UK medical education, while also underscoring the cultural and practical challenges of doing so. Future research could explore whether formal training in feedback skills leads to doctors who use feedback more effectively and whether this improves patient outcomes. It may also be useful to examine how such frameworks function in more egalitarian healthcare cultures, such as the UK.

I sincerely appreciate the author’s insightful remarks on improving medical education at an undergraduate level.

Imperial College London is grateful for support from the NIHR Applied Research Collaboration NW London. The views expressed in this publication are those of the authors and not necessarily those of the NIHR or the Department of Health and Social Care.

## Data Availability

No new data were generated or analysed for this Letter to the Editor.
